# Azole Resistance in Clinical and Environmental *Aspergillu*s Isolates from the French West Indies (Martinique)

**DOI:** 10.3390/jof7050355

**Published:** 2021-04-30

**Authors:** Lorra Monpierre, Nicole Desbois-Nogard, Isabel Valsecchi, Marielle Bajal, Cécile Angebault, Charline Miossec, Françoise Botterel, Éric Dannaoui

**Affiliations:** 1 Unité de Parasitologie-Mycologie, Département Prévention, Diagnostic, Traitement des Infections, CHU Henri Mondor, Assistance Publique des Hôpitaux de Paris (AP-HP), 94140 Créteil, France; lorra.monpierre@hotmail.fr (L.M.); cecile.angebault@aphp.fr (C.A.); francoise.botterel@aphp.fr (F.B.); 2 EA DYNAMiC 7380, Faculté de Santé, Univ Paris-Est Créteil (UPEC), Créteil, France-École Nationale Vétérinaire d’Alfort (ENVA), USC Anses, 94700 Maison-Alfort, France; isabel.valsecchi@u-pec.fr; 3 Laboratoire de Parasitologie-Mycologie Hôpital Pierre Zobda-Quitman, CHU de la Martinique, 97261 Fort-de-France, France; nicole.desbois-nogard@chu-martinique.fr (N.D.-N.); marielle.bajal@gmail.com (M.B.); charlinemiossec@gmail.com (C.M.); 4 Unité de Parasitologie-Mycologie, Service de Microbiologie, Hôpital Européen Georges Pompidou, AP-HP, 75015 Paris, France; 5 Université de Paris, 75006 Paris, France

**Keywords:** azole resistance, West Indies, Martinique, *Aspergillus fumigatus*, *Aspergillus terreus*, *cyp51A* gene mutations

## Abstract

The emergence of azole resistant *Aspergillus* spp., especially *Aspergillus fumigatus*, has been described in several countries around the world with varying prevalence depending on the country. To our knowledge, azole resistance in *Aspergillus* spp. has not been reported in the West Indies yet. In this study, we investigated the antifungal susceptibility of clinical and environmental isolates of *Aspergillus* spp. from Martinique, and the potential resistance mechanisms associated with mutations in *cyp51A* gene. Overall, 208 *Aspergillus* isolates were recovered from clinical samples (*n* = 45) and environmental soil samples (*n* = 163). They were screened for resistance to azole drugs using selective culture media. The Minimum Inhibitory Concentrations (MIC) towards voriconazole, itraconazole, posaconazole and isavuconazole, as shown by the resistant isolates, were determined using the European Committee on Antimicrobial Susceptibility Testing (EUCAST) microdilution broth method. Eight isolates (*A. fumigatus*, *n* = 6 and *A. terreus*, *n* = 2) had high MIC for at least one azole drug. The sequencing of *cyp51A* gene revealed the mutations G54R and TR34/L98H in two *A. fumigatus* clinical isolates. Our study showed for the first time the presence of azole resistance in *A. fumigatus* and *A. terreus* isolates in the French West Indies.

## 1. Introduction

Acquired azole resistance in *Aspergillus* spp. was first reported in the late 1990s [1,2] and has since become a widespread problem [3] usually associated with a higher rate of treatment failure necessitating therapeutic adjustment in aspergillosis [4,5]. This resistance concerns mainly *Aspergillus fumigatus* [6], though it has also involved other species, such as *Aspergillus flavus* [7] and *Aspergillus terreus* [8,9,10]. The prevalence of such a resistant species varies in the world, and several studies have shown high rates, particularly in the Netherlands (0.8–29%), the United Kingdom (6.6–27.8%), and Germany (3.2–30%) [3,11].

Two routes of resistance acquisition have been identified: (i) the natural in vivo emergence of resistant isolates in aspergillosis patients subjected to long-term azoles treatment (patient-acquired route), and (ii) the development of resistant isolates in the environment (environment-acquired route) [12,13,14]. The environmental route of azole resistance development has been reported since the late 2000s [15] and is largely responsible for the global emergence of resistance. The intensive use of azole fungicides in the agriculture industry expose *Aspergillu*s spp. in the environment to azole fungicides and induce cross-resistance to medical azoles [13,16,17].

For *A. fumigatus*, the most commonly reported mechanism of resistance development is the alteration of *cyp51A* gene which codes for 14-alpha-demethylase, the target enzyme of azole drugs [12]. Several studies demonstrated that the type of *cyp51A* mutations depends on the route of resistance acquisition. In clinical isolates, the main resistance-development mechanisms are the substitution of amino acids in position G54, G138, or M220 of CYP51A protein [18,19,20]. More recently, substitutions of CYP51A protein amino acids, combined with a tandem repeat in the promoter region of *cyp51A* gene (TR34/L98H, TR46/Y121F/T289A, or rarely TR53), have been reported worldwide in azole-naïve patients and in the environment [15,21], which suggests an environmental route of resistance.

In the French West Indies (Guadeloupe and Martinique), using triazole fungicides (difeconazole and propiconazole) is allowed in agriculture, especially in banana plantations, to eliminate *Mycosphaerella fijiensis*, a phytopathogenic fungus that causes Sigatoka disease [22]. The use of these fungicides is under strict control allowing a maximum of 6 to 8 rounds of treatment per year in order to limit their environmental impact. Considering the risk of emergence of azole fungicides-resistant species in Martinique, the aim of the present study was to analyze the prevalence of azole resistance in clinical and environmental *Aspergillus* isolates in the French West Indies, and to determine whether *cyp51A* gene alterations were responsible for the emergence of such resistance.

## 2. Materials and Methods

### 2.1. Clinical Samples

All phenotypically identified *Aspergillus* spp. detected in different samples drawn from patients hospitalized in Pierre Zobda-Quitman hospital (Martinique University Hospital, Fort-de-France, West Indies, France) from January 2014 to December 2018 were analyzed. Each isolate was stored at −80 °C before culture on Malt-extract agar (VWR, Fontenay-sous-Bois, France).

### 2.2. Environmental Samples

**Figure 1:**
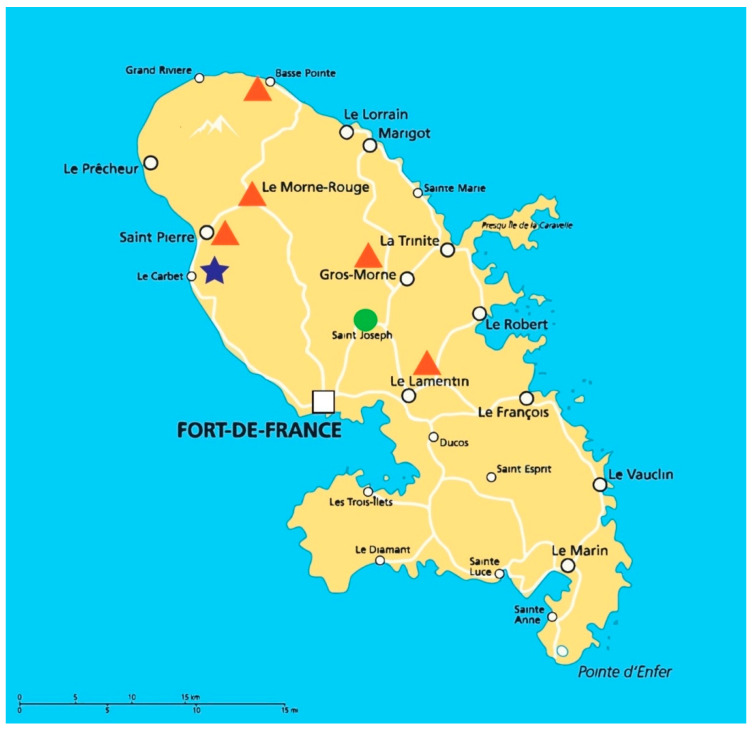
Geographical localization of soil samplings. The orange triangles represent the localization of banana plantations, the blue star, the sugar cane field and the green circle, the natural forest.

A total of 140 soil samples were collected from northern and central regions of Martinique, of which 100 came from five banana plantations (Banamart^®^; at Lamentin, Gros-Morne, Morne-Rouge, Basse-Pointe, and Saint-Pierre, Martnique,), 20 from one sugar cane field (Neisson^®^; at Le Carbet, Martinique) and 20 from a natural forest (Saint Joseph, Martinique) (Figure 1).

The samples were treated as previously described [15] with few modifications. Briefly, 2 g of soil was suspended in sterile distilled water with 1% Tween-20 (Sigma-Aldrich, Saint-Quentin Fallavier, France). Fifty microliters of the suspension was plated on Malt-extract agar (VWR) supplemented with 0.5 g/L of chloramphenicol (Sigma-Aldrich) and incubated at 37 °C for 48 h. *Aspergillus* spp. isolates were phenotypically identified using macroscopic and microscopic characteristics.

### 2.3. Screening for Azole Resistance

Screening for azole resistance was performed as previously described [23,24,25] using 3-well plates containing RPMI-1640 agar medium (Sigma-Aldrich, Saint-Quentin-Fallavier, France); the first well was supplemented with 4 mg/L itraconazole (Sigma-Aldrich), the second with 1 mg/L voriconazole (Sigma-Aldrich), and the third was without drug (growth control). Although this method has been validated for the detection of azole resistance only in Aspergillus fumigatus [26,27], it has been used in previous studies for other Aspergillus species [28,29]. Therefore, in this study, we attempted to use this method for screening azole resistance also in non-fumigatus Aspergillus species in order to explore its utility for these other species. Each well was inoculated with conidia from a colony of Aspergillus isolates, and plates were incubated at 37 °C for 48 h. Two independent readers assessed the results qualitatively as negative (no visible growth) or positive (presence of fungal colonies). A. fumigatus ATCC 13073 and an azole-resistant A. fumigatus were used as susceptible and resistant controls, respectively.

### 2.4. Antifungal Susceptibility Testing

Isolates detected as resistant by the screening method were further tested for antifungal susceptibility using the European Committee for Antimicrobial Susceptibility Testing (EUCAST) microdilution broth reference method [30]. All clinically available azole antifungal molecules were tested: itraconazole (ITZ) (Sigma-Aldrich); voriconazole (VCZ) (Sigma-Aldrich); posaconazole (PCZ) (MSD, Kenilworth, NJ, USA); and isavuconazole (ISA) (Basilea Pharmaceutica International Ltd., Basel, Switzerland). The number of conidia inoculated was 10^5^ to 2.5 × 10^5^ cfu/mL and the concentrations of each drug ranged from 0.016 to 8 mg/L. After 48 h of incubation at 37 °C, the minimum inhibitory concentration (MIC) was determined, both visually, as the lowest drug concentration that could completely inhibit fungal growth, and spectrophotometrically at 550 nm using a 90% growth inhibition endpoint. All tests were performed in triplicate. *Candida parapsilosis* ATCC 22019 and *Candida krusei* ATCC 6258 were included as quality controls.

### 2.5. Interpretation of MIC

*A. fumigatus* resistance was defined according to the EUCAST clinical breakpoints, i.e., at concentrations of >1 mg/L for ITZ and VCZ, >2 mg/L for ISA, and >0.25 mg/L for PCZ. For *A. terreus*, breakpoints were similar except for ISA (>1 mg/L), and for VCZ for which Epidemiological Cut off Value (ECV) of >1 mg/L was used as breakpoints.

### 2.6. Molecular Identification

Resistant isolates detected by screening and EUCAST microdilution broth reference methods were identified by sequencing a portion of the β-tubulin gene [31]. Complete genomic DNA was extracted from a Malt-extract agar fungal culture using QIAamp DNA blood minikit (Qiagen Sciences Ing, Courtaboeuf, France). Briefly, conidia and hyphae were disrupted with glass beads (VWR, ref: 432-0064) and lysis buffer on MagNA Lyser instrument (Roche Diagnostics, Meylan, France). The resulting suspension was then treated according to the manufacturer’s instructions. PCRs were performed in a 50 µL-final volume containing 1× HF buffer (ThermoFisher, Les Ulis France), 200 µM of deoxynucleoside triphosphates (dNTPs), 1 µM of each primer, 3% of DMSO, 1 unit of Phusion™ High-Fidelity DNA Polymerase (ThermoFisher), and 100 ng of genomic DNA. The primers used for β-tubulin gene amplification were Bt2a (5′-GGTAACCAAATCGGTGCTGCTTTC-3′) and Bt2b (5′-ACCCTC AGTGTAGTGACCCTTGGC-3′) as previously described [32]. Sanger sequencing was performed at the Genomic platform of Henri Mondor Hospital Biomedical Research Institute (IMRB). Sequences were analyzed using DNA Baser Assemble v5.15.0 and compared to GenBank and MycoBank databases sequences. Nucleotide identification was achieved at >99% match.

### 2.7. cyp51 Gene Sequencing

For azole-resistant isolates, the whole *cyp51A* gene (14-alpha sterol demethylase) and its promoter region were Sanger sequenced to detect azole resistance-causing alterations. PCRs were performed in a 50 µL-final volume containing 1X HF buffer (ThermoFisher), 200 µM of deoxynucleoside triphosphates (dNTPs), 1 µM of each primer, 3% of DMSO, 1 unit of Phusion™ High-Fidelity DNA Polymerase (ThermoFisher), and 100 ng of genomic DNA. PCR products were Sanger sequenced using primers as previously described [8,9,33,34]. DNA sequences electropherograms were analyzed with DNA Baser Assembler v5.15.0 and CLC Sequence Viewer 8.0 software. The sequences were compared with two standard *cyp51A* genes: AFUB_063960 of *A. fumigatus* reference strain CBS144, and ATEG_05917 of *A. terreus* reference strain NIH2624.

### 2.8. Ethical Approval

All procedures contributing to this work are in compliance with the ethical standards of the Helsinki Declaration of 1975, as revised in 2008. The study was retrospectively conducted on isolates collected through routine clinical work and patients’ identifiable information had already been anonymized; no written or verbal informed consent was necessary for patients to participate in this study.

## 3. Results

### 3.1. Clinical Isolates

#### 3.1.1. Culture and Morphological Identifications

During the study period, 45 clinical *Aspergillus* spp. isolates were collected from bronchoalveolar lavages (*n* = 14), bronchial fluids (*n* = 7), sinus samples (*n* = 7), sputa (*n* = 5), pleural fluids (*n* = 4), ear (*n* = 5), cornea (*n* = 2), and nose samples (*n* = 1) of 39 patients hospitalized at Pierre Zobda-Quitman hospital (Martinique University Hospital, Fort-de-France, West Indies, France). These isolates were first morphologically identified as *A. fumigatus sensu lato* (*n* = 35, 78%), *A. flavus sensu lato* (*n* = 8, 18%) and *A. terreus sensu lato* (*n* = 2, 4%) (Table 1).

#### 3.1.2. Antifungal Susceptibilities

Screening on RPMI-agar plates supplemented with azoles identified 16 potentially resistant isolates (Figure S1): *A. fumigatus* (*n* = 10), *A. flavus* (*n* = 4) and *A. terreus* (*n* = 2). Using the EUCAST microdilution method, the MIC of at least one azole was high in only six of such isolates (four of *A. fumigatus* and two of *A. terreus*) (Table 2).

#### 3.1.3. Molecular Identification

Identification of the species level of resistant isolates was confirmed by sequencing of part of β-tubulin gene. The four resistant *A. fumigatus* were of *A. fumigatus stricto sensu* (ss) species, and the two resistant *A. terreus* were of *A. terreus stricto sensu* (ss) species.

#### 3.1.4. cyp51A Gene Sequencing

The analysis of DNA sequences revealed the presence *cyp51A* alterations in two azole-resistant *A. fumigatus* isolates (Figure S1). The first isolate (Af 003) harbored L98H mutation with a 34 bp tandem repeat in its promoter region (TR34/L98H), and the second (Af 002) had G54R mutation (Table 2). No alterations in the cyp51A gene were found in the remaining four resistant isolates (Table 2).

### 3.2. Environmental Isolates

#### 3.2.1. Culture and Morphological Identifications

The 163 *Aspergillus* species isolated from the 140 soil samples were *A. fumigatus sensu lato* (*n* = 98; 70%), *A. flavus sensu lato* (*n* = 43, 26%), *A. terreus sensu lato* (*n* = 19, 12%), and other species (*n* = 3, 2%) (Table 1).

#### 3.2.2. Antifungal Susceptibilities

Screening on RPMI-agar plates supplemented with azoles identified 51 potentially resistant isolates (Figure S2): *A. fumigatus* (*n* = 36), *A. flavus* (*n* = 13) and *A. terreus* (*n* = 2). Among these isolates, only two *A. fumigatus* showed high MICs of VCZ (2–4 mg/L), ITZ (2 mg/L), PCZ (0.5 mg/L), and ISA (2–4 mg/L). One of these isolates (Af 005) was collected from a banana plantation in Lamentin, and the other (Af 006) from the sugar cane field in Le Carbet (Table 3).

#### 3.2.3. Molecular Identification

Identification of these resistant isolates as *A. fumigatus sensu stricto* was confirmed by sequencing of part of β-tubulin gene.

#### 3.2.4. cyp51A Gene Sequencing

No alterations in *cyp51A* gene were found in these two resistant isolates (Figure S2).

## 4. Discussion

The emergence of azole-resistant *Aspergillus* species is a public health problem affecting many regions worldwide with varying levels of prevalence [3]. In France, and according to the studied population, such a prevalence has been evaluated at less than 10% [23,34,35]. Our present study, which was carried out in the French West Indies, reveals a high level of azole-resistant *Aspergillus* in patients and the appearance of few azoles-resistant environmental strains. Due to the small number of isolates studied, we could not determine a precise prevalence of resistance, but we determined approximate rates.

In clinical isolates of *Aspergillus* spp., the rate of azole resistance was 13% (6/45) in an unselected patients’ population over a 4-year period (2014–2018). This prevalence is higher than that observed in other French studies, even those involving cystic fibrosis cases, known to have a high prevalence of azole-resistant *Aspergillus* [35]. Both *A. fumigatus* (*n* = 4) and *A. terreus* (*n* = 2) isolates were concerned. On the other hand, in the environment, the rate of azole resistance in *Aspergillus* spp. was low, around 1%, and concerned only *A. fumigatus* (*n* = 2). These resistant isolates were collected from two agricultural plantations, one from a banana plantation located in the center of the island (Lamentin) and the other from a sugar cane field in the north of the Island (Le Carbet).

Two routes of resistance acquisition in *Aspergillus* have been identified (i) an in vivo selection of resistant isolates as a consequence of long-term treatment with medical azoles in patients with *Aspergillus* diseases (patient-acquired route) and (ii) an acquisition of resistant isolates directly from the environment related to the use of azoles fungicides in agriculture. In Martinique, we found a higher rate in patients than in the environment. This could be related to an important use of azoles drugs as preventive or curative treatment in patients. We do not have this information because the study was retrospectively conducted on isolates collected through routine clinical work patients’ information and data were anonymized.

Previous reports revealed high resistance rates in clinical and environmental *A. fumigatus* isolates in the Netherlands, UK, and Germany [3,11]. In the aforementioned countries, azole fungicides are extensively used in agriculture to protect grains, fruits, vegetables, or flowers crops from mildew infection [36]. In the French West Indies, several strategies have been adopted in the past few years to limit the environmental impact of fungicides used in banana plantations. Such strategies focused on reducing the frequency of fungicide application in the plantations, regularly clearing infected leaves, or using paraffin oil instead of chemical fungicides whenever feasible. Although the number of isolates studied is too small to draw firm conclusions, we can hypothesize that these measures could explain the low prevalence of azole resistant *A. fumigatus* in banana plantations as observed in our study. A larger study involving more isolates should be performed to confirm this hypothesis. On the other hand, we do not know how to explain the presence of a resistant isolate in the sugar cane field because it is an organic plantation that does not receive antifungal treatment.

The most commonly reported mechanism of resistance development is the alteration of the target enzyme of azole drugs, namely lanosterol 14α-demethylase (CYP51). *Aspergillus* spp. contain two or three CYP51 isoenzyme: CYP51A, CYP51B or CYP51C depending on the species [37]. However, the main azole resistance mechanism is the development of point mutations in *cyp51A* gene. In this study, the sequencing of *cyp51A* gene of resistant *Aspergillus* spp. isolates showed alterations in two clinical *A. fumigatus* isolates; one harbored G54R mutation and the other carried TR34/L98H insertion/mutation. Both alterations are well known to confer azole resistance in *A. fumigatus* [38]. More specifically, substituting glycine, the smallest amino acid, for arginine, a larger amino acid, at position 54, has been described as a mechanism to prevent the access of long chain azoles, such as ITZ and PCZ, to the active site on CYP51A protein, thereby reducing the affinity of drug-enzyme interaction [39]. Our results are in agreement with this fact as isolate Af 002 which harbored G54R mutation showed high MICs (>8 mg/L) for ITZ and PCZ, but low MIC for VCZ (0.5 mg/L) and ISA (1 mg/L). Amino acid substitutions at position G54 were first recognized in patients on long-term azole therapy. However, recent publications have also shown the presence of these substitutions in environmental isolates. G54E mutation was reported in environmental samples in Romania, India, and Tanzania [40], G54A in Germany [41], and G54R in Switzerland [21]. The combination of a 34-bp tandem repeat sequence in the promoter gene of *cyp51A* gene with the substitution of leucine 98 for histidine, in the protein, is a common mechanism widely described worldwide [12]. The latter appears to be an environmentally acquired alteration and is not related to selective pressure due to prior azole treatment in patients [13,15,42].

Among the six azole-resistant *A. fumigatus* found in this study, four isolates (two from patients’ samples and two from the environment) showed no mutations in *cyp51A* gene or in its promotor, suggesting other molecular mechanisms. Although azole resistance in *A. fumigatus* is mainly attributed to *cyp51A* gene mutations, other resistance mechanisms have been previously reported and increasingly brought to light over the past years [43,44]. The most common of these mechanisms is the overexpression of efflux pumps, ATP-binding cassette proteins (ABC) or major facilitator superfamily pumps (MFS), which are employed in eukaryotic organisms for cell detoxification. Overexpression of these efflux pumps reduces the azole drug concentrations inside fungal cells, hence the resistance. In *A. fumigatus*, at least 49 ABC and 278 MFS transporter genes have been described, but to date only a few genes are known to be linked to azole resistance, including AfuMDR1, AfuMDR2, AfuMDR3, AfuMDR4, AbcA-E, MfsA-C, and AtrF [45,46]. Among other mechanisms correlated to azole resistance, Camps et al. described in 2012 using whole genome sequencing, a P88L substitution in CCAAT-binding transcription factor HapE in a clinical *Aspergillus fumigatus* isolate [47]. Other mutations/alterations in other genes/proteins have also been documented [48,49,50,51,52,53].

The two clinical isolates of *A. terreus* detected in this study showed an MIC greater than the ECV established for VCZ and an MIC greater than the breakpoint established for ITZ, PCZ and ISA. In *A. terreus*, substitutions in CYP51A at position M217 were previously associated with reduced susceptibility to ITZ (MIC of 1.0–2.0 μg/mL), VCZ (MIC of 1.0–4.0 μg/mL) and PCZ (MIC of 0.25–0.5 μg/mL) [8]. However, this mutation was not identified in our isolates. Other azole-resistant *A. terreus* isolates with wild-type CYP51A were previously reported, but no other alterations have been described to explain their resistance yet [9,10].

The next step in our work will be to perform whole genome sequencing of isolates from our study in order to compare their genetic background, to detect possible new resistance-inducing mechanisms, and to perform a phylogenetic analysis that related the origin of the isolates.

In conclusion, we showed herein for the first time the presence of azole resistance in *A. fumigatus* and *A. terreus* isolates in Martinique, with a higher prevalence in clinical isolates than in environmental isolates. Our results contribute to the overall knowledge of the epidemiology of *Aspergillus* resistance in the world and give a clearer image of the prevalence of azole resistance in the French West Indies. Further surveillance of azole resistance both in patients and in the environment of West Indies is warranted.

## Figures and Tables

**Table 1:** Species distribution of *Aspergillus* spp. recovered from clinical and environmental samples.

Sample Source	*N*	*A. fumigatus*	*A. flavus*	*A. terreus*	Other *Aspergilli*
		*n* (%)	*n* (%)	*n* (%)	*n* (%)
Clinical	45	35 (78)	8 (18)	2 (4)	0 (0)
Environmental	163	98 (60)	43 (26)	19 (12)	2 (2)

**Table 2:** Characteristics and in vitro antifungal susceptibility profiles of azole-resistant *Aspergillus* spp. isolates (*n* = 6) from clinical samples.

Isolate	Species	Origin	Sample Type	MIC (mg/L)				*cyp51A* Gene
				VCZ	ITZ	PCZ	ISA	
Af 001	*A. fumigatus*	Patient	BAL ^1^	2	1	0.25	1	WT ^3^
Af 002	*A. fumigatus*	Patient	BAL ^1^	0.5	>8	>8	1	G54R
Af 003	*A. fumigatus*	Patient	BAL ^1^	4	>8	1	8	TR34/L98H
Af 004	*A. fumigatus*	Patient	BAL ^1^	2	2	1	4	WT ^3^
At 001	*A. terreus*	Patient	BA ^2^	>8	1	1	>8	WT ^3^
At 002	*A. terreus*	Patient	BA ^2^	2	1	0.5	4	WT ^3^

^1^ BAL: Broncho-alveolar lavage; ^2^ BA: Bronchial aspiration; ^3^ WT: Wild-Type.

**Table 3:** Characteristics in vitro antifungal susceptibility profiles of azole-resistant *Aspergillus* spp. isolates (*n* = 2) from environmental samples.

Isolate	Species	Origin	Sample Type	MIC (mg/L)				*cyp51A* Gene
				VCZ	ITZ	PCZ	ISA	
Af 005	*A. fumigatus*	Banana plantation	Soil sample	2	2	0.5	4	WT ^1^
Af 006	*A. fumigatus*	Sugar cane field	Soil sample	4	2	0.5	2	WT ^1^

^1^ WT: Wild-Type.

## Data Availability

Data is contained within the article or supplementary material.

## Supplementary Materials

The following are available online at https://www.mdpi.com/article/10.3390/jof7050355/s1, Figure S1: Workflow of analyses performed on clinical isolates; Figure S2: Workflow of analyses performed on environmental isolates.
