# Supplementary figures and images for: Azole Resistance in Clinical and Environmental Aspergillus Isolates from the French West Indies (Martinique)
Source: J Fungi (Basel). 2021 Apr 30;7(5):355. doi: 10.3390/jof7050355 (PMC8147181; doi:10.3390/jof7050355)

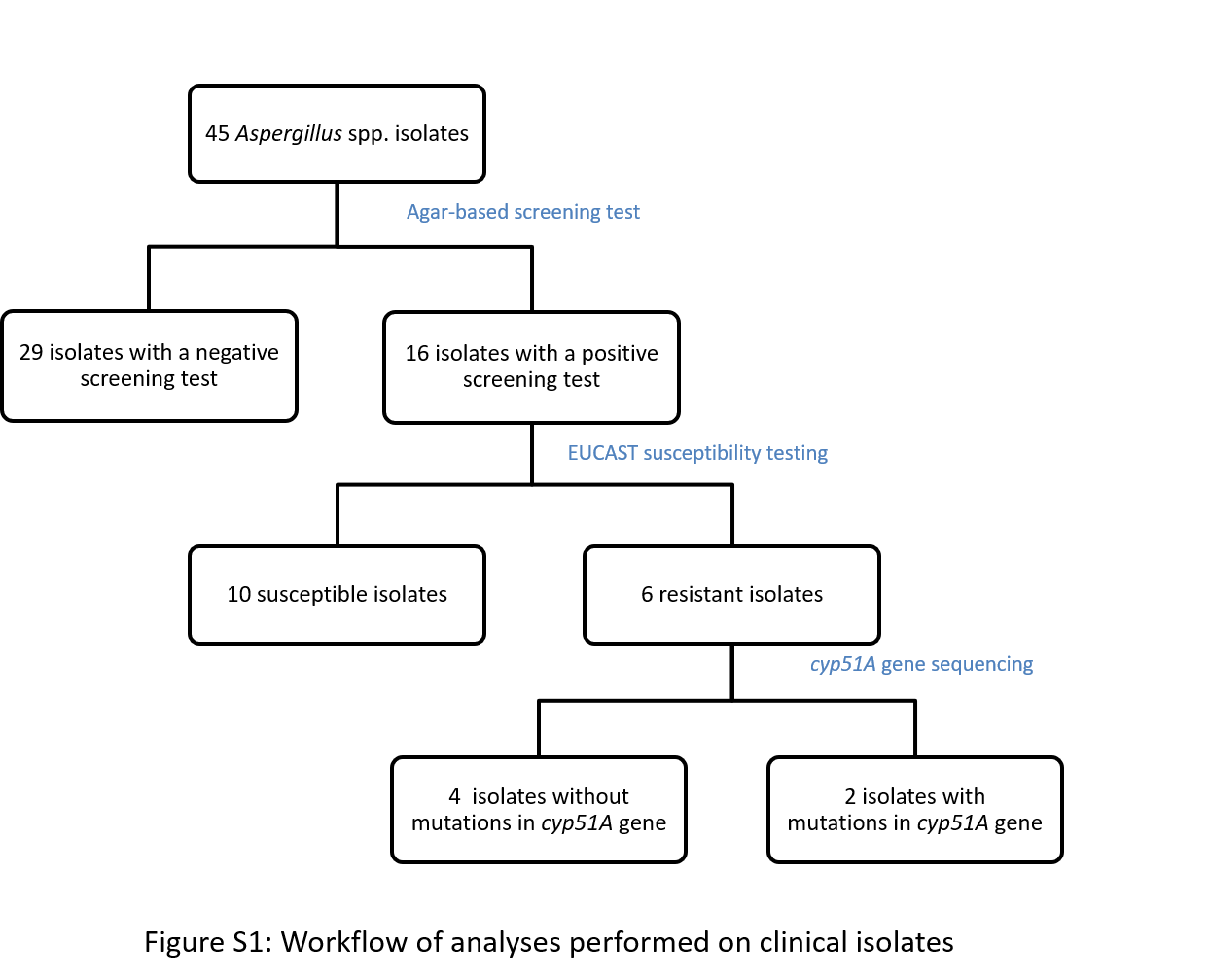

Supplement: Supplementary file 1 [file jof-07-00355-s001.zip › jof-1153255-figure s1.png]

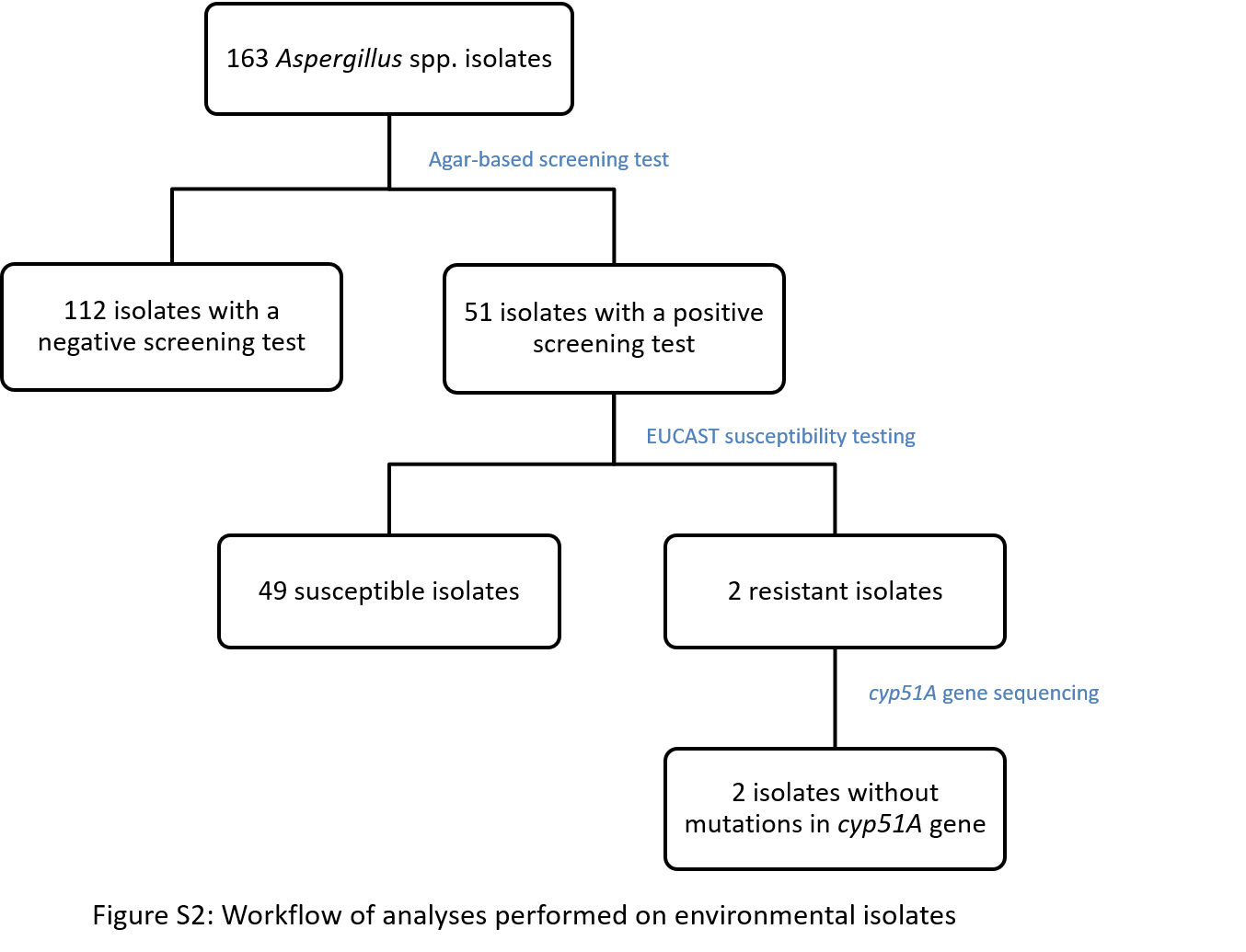

Supplement: Supplementary file 1 [file jof-07-00355-s001.zip › jof-1153255-figure s2.png]
